# Artificial liver plasma adsorption improves survival in elderly patients with severe pneumonia: a retrospective cohort study

**DOI:** 10.3389/fmed.2025.1613810

**Published:** 2025-07-09

**Authors:** Richai Chen, Jiaqian Jin, Sainan Zhang, Jiajun Wu, Qiangqiang Xiang, Xuanhao Lin, Danhua Zhu, Mengfei Zhu

**Affiliations:** ^1^Key Laboratory of Artificial Organs and Computational Medicine in Zhejiang Province, Shulan (Hangzhou) Hospital Affiliated to Shulan International Medical College, Zhejiang Shuren University, Hangzhou, China; ^2^Zhejiang Chinese Medical University, Hangzhou, Zhejiang, China; ^3^State Key Laboratory for Diagnosis and Treatment of Infectious Diseases, National Clinical Research Center for Infectious Diseases, National Medical Center for Infectious Diseases, Collaborative Innovation Center for Diagnosis and Treatment of Infectious Diseases, The First Affiliated Hospital, Zhejiang University School of Medicine, Hangzhou, Jiangsu, China

**Keywords:** artificial liver, elderly patients, severe COVID-19 pneumonia, cytokine storm, interleukin-6

## Abstract

**Objective:**

To assess the therapeutic efficacy of artificial liver plasma adsorption (ALPA) in patients with severe pneumonia.

**Methods:**

This retrospective study enrolled 151 patients meeting severe pneumonia diagnostic criteria who were admitted to the intensive care unit at Shulan Hospital, Hangzhou, China, between January 2020 and December 2024. Participants were allocated to either: (1) the ALPA intervention group (*n* = 56) receiving artificial liver plasma adsorption (ALPA) therapy, or (2) the control group (*n* = 95) receiving standard treatment. This study prospectively collected comprehensive clinical data, including: (1) baseline demographic characteristics; (2) serial measurements of laboratory parameters (e.g., white blood cell count (WBC), lymphocyte percentage (LY%), C-reactive protein (CRP), procalcitonin (PCT), and interleukin-6 (IL-6)) at pre- and post-treatment intervals; and (3) Pneumonia Severity Index (PSI) scores at admission. Patient survival outcomes were systematically recorded, including: (1) time-to-event endpoints (overall survival duration from enrollment to death or last follow-up), and (2) clinical outcomes.

**Results:**

No statistically significant differences were observed between the two groups regarding WBC, CRP, PCT, or IL-6 levels at baseline (all *p* ≥ 0.05). However, the LY% in the artificial liver treatment group was significantly lower compared to the conventional therapy group (*p* < 0.05). Post-treatment analysis revealed that the ALPA group demonstrated significantly lower levels of WBC, LYM, CRP, PCT, and IL-6 compared to control group (*p* < 0.05). The control group exhibited significant post-treatment elevations in WBC (*p* < 0.05), whereas LY%, PCT, IL-6, and CRP levels showed no significant variation (*p* ≥ 0.05). In the ALPA group, WBC, LY%, and PCT levels remained stable (*p* ≥ 0.05), while CRP, IL-6 demonstrated a significant reduction (*p* < 0.05). Post-treatment mortality rates differed significantly between groups (42.9% in ALPA group versus 81.1% in controls; *p* < 0.001). The treatment group showed a 38.2% relative reduction in mortality compared to controls, achieving statistical significance (*p* < 0.001). Multivariable Cox proportional hazards regression demonstrated that both elevated PSI Score at hospital admission (adjusted HR = 1.009, *p* = 0.006) and ALPA treatment (adjusted HR = 4.134, *p* < 0.001) independently predicted poorer survival outcomes. ALPA treatment was associated with significantly improved survival outcomes compared to controls (mean: 218.42 vs. 36.81 days; median not reached vs. 27.0 days; HR = 0.1907, *p* < 0.0001 by log-rank test).

**Conclusion:**

Artificial liver plasma adsorption (ALPA) therapy demonstrates significant clinical efficacy by effectively suppressing inflammatory markers (e.g., IL-6, CRP) and attenuating cytokine storm progression. This treatment significantly reduces mortality and prolongs survival time in elderly patients with severe pneumonia.

## Introduction

Geriatric severe pneumonia (SP) is a life-threatening condition characterized by diffuse alveolar exudation caused by bacterial, viral, or fungal pathogens, which may progress to multiple organ failure. The pathogenesis involves: (1) pathogen invasion with host defense imbalance, (2) excessive inflammatory response leading to cytokine storm, (3) alveolar-capillary barrier disruption, (4) secondary immunosuppression with opportunistic infections, (5) coagulation abnormalities and microthrombosis, and (6) multiple organ dysfunction syndrome (MODS) (1–4). Current evidence establishes cytokine storm syndrome (CS) as a critical link between acute respiratory distress syndrome (ARDS), septic shock (SS), and MODS (3), and a key pathogenic mechanism in severe COVID-19 (5).

ALPA is an extracorporeal therapy developed to address the pathophysiological mechanisms underlying liver failure. Through decades of clinical advancement, ALBPS has evolved into a versatile intervention with applications extending beyond hepatic disorders, including cytokine storm (CS) management (6). During the H7N9 avian influenza pandemic, ALPA combined with extracorporeal membrane oxygenation (ECMO) demonstrated clinical efficacy by attenuating cytokine storms and ameliorating both ARDS and MODS in critically ill patients (7). In COVID-19 patients, ALPA treatment yielded significant reductions in disease severity scores (APACHE II, PSI, and SOFA) and cytokine levels, correlating with marked clinical improvement (8).

Severe pneumonia, particularly in geriatric populations, presents complex pathophysiology and rapid disease progression, often lacking rapid and effective treatment strategies. While preliminary studies suggest that ALPA may demonstrate therapeutic efficacy in patients with severe/critical viral pneumonia (9), limitations including small sample sizes and insufficient data preclude definitive conclusions regarding its clinical impact on elderly patients with severe/critical pneumonia, particularly in terms of mortality outcomes.

This study aimed to assess mortality outcomes and laboratory parameter variations in geriatric patients with severe pneumonia, while conducting a preliminary therapeutic efficacy evaluation.

## Methods

### Study population and grouping

This retrospective study enrolled geriatric patients with severe pneumonia admitted to the ICU of Shulan (Hangzhou) Hospital between January 2020 and December 2024. The etiological profile included viral, bacterial, fungal and mixed infections. Severe pneumonia diagnosis complied with the 2016 Chinese Guidelines for Diagnosis and Treatment of Adult Community-Acquired Pneumonia (1). The study used the following selection criteria:

### Inclusion criteria

1. Severe pneumonia diagnosis in accordance with the 2016 Chinese Guidelines for Diagnosis and Treatment of Adult Community-Acquired Pneumonia: The diagnosis of severe pneumonia can be established when a patient meets either one major criterion or three or more minor criteria. The major criteria include: (1) requirement for endotracheal intubation and mechanical ventilation; and (2) septic shock necessitating vasopressor therapy despite adequate fluid resuscitation. The minor criteria consist of: (1) respiratory rate ≥30 breaths per minute; (2) ratio of arterial oxygen partial pressure to fractional inspired oxygen (PaO2/FiO2) ≤250 mmHg (1 mmHg = 0.133 kPa); (3) multilobar infiltrates on imaging; (4) altered mental status and/or disorientation; (5) blood urea nitrogen level ≥7.14 mmol/L; and (6) systolic blood pressure <90 mmHg requiring aggressive fluid resuscitation.
2. Age ≥ 55 years.

### Exclusion criteria

1. Hospitalization duration <48 h.
2. Other ineligibility factors (e.g., incomplete medical records).

The study initially screened 175 cases, with 151 patients meeting the inclusion criteria and providing complete clinical data. Of the 24 excluded cases, 10 resulted from patient deaths within 48 h post-admission, and 14 were excluded due to incomplete medical records (10% of total cases). Patients were stratified into two treatment groups based on ALPA administration: the control group (*n* = 95) receiving standard care alone, and the ALPA group (*n* = 56) receiving combined plasma adsorption and standard therapy. This retrospective study complied with the Declaration of Helsinki (2013 revision) ethical guidelines.

### Clinical data and laboratory parameters

Demographic characteristics, including sex, age, height, and weight, were recorded for all patients. Laboratory parameters such as white blood cell count (WBC), lymphocyte percentage (LY%), C-reactive protein (CRP), interleukin-6 (IL-6), and procalcitonin (PCT) were assessed before treatment and within 1 week post-treatment. Patients who died during hospitalization or were discharged against medical advice (DAMA) were classified as having mortality outcomes, while those with clinical improvement were categorized as non-mortality events. The study dataset contained a small proportion of missing values, all of which were determined to be either missing completely at random (MCAR) or missing at random (MAR). Samples with >10% missing data were excluded from analysis, while those with <10% missing values were imputed using the “unknown” category.

### Treatment protocols

**Figure 1:**
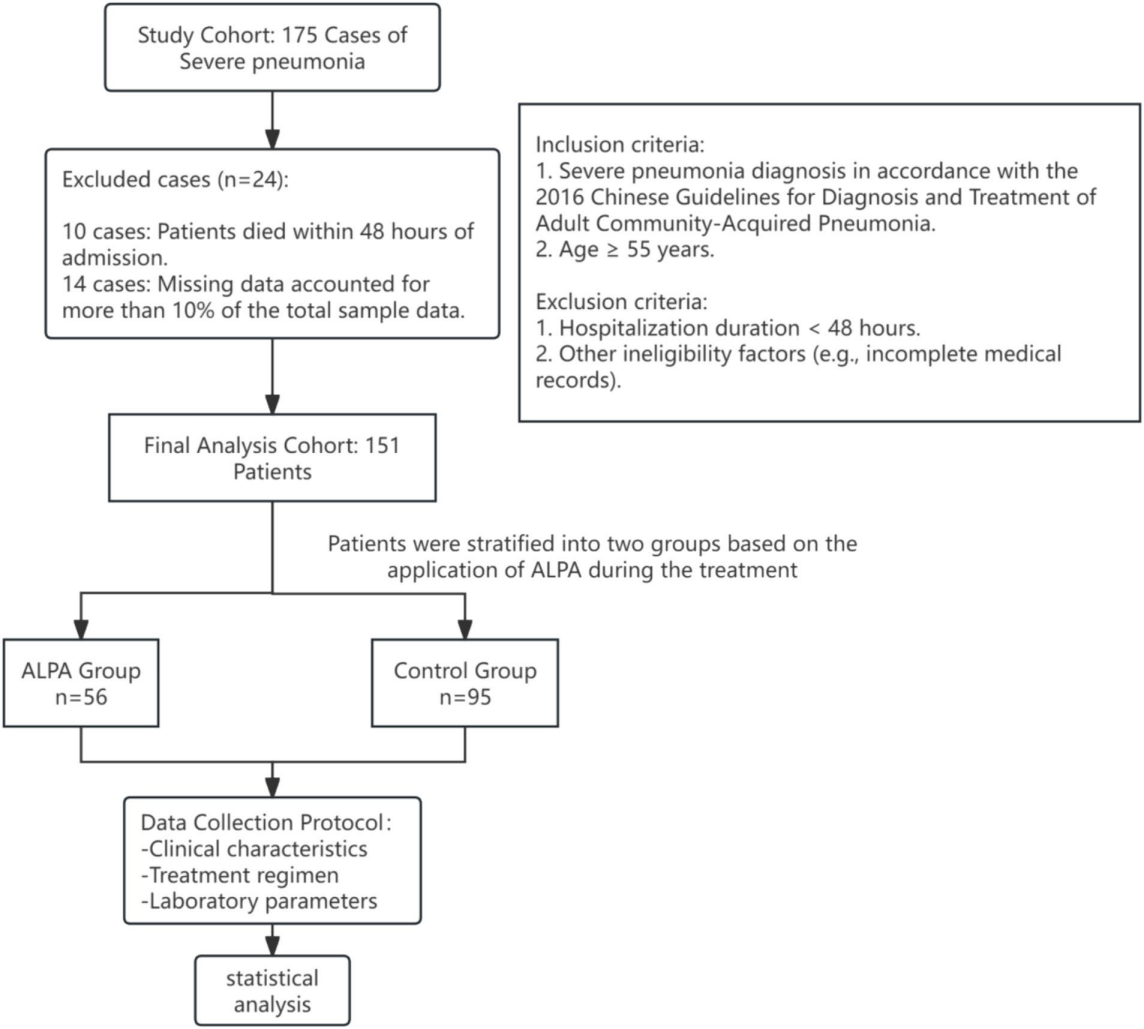
Technical roadmap.

The ALPA treatment group received resin-based plasma adsorption combined with comprehensive internal medicine therapy, while the control group received standard internal medicine treatment alone. Both groups received methylprednisolone (0.8 mg/kg/day) (10) and targeted antimicrobial therapy including antiviral, antibacterial, and antifungal agents during treatment. ALSS was initiated according to consensus guidelines (6) when patients exhibited: (1) acute organ dysfunction, or (2) serum IL-6 levels ≥5 times the upper normal limit (UNL) or daily increase ≥1 UNL. Treatment efficacy was assessed by post-procedure IL-6 reduction ≥25% from baseline or normalization to ≤2 UNL. Patients failing to meet these criteria should undergo prompt reassessment for ALPA indication. Conversely, ALPA indication assessments may be deferred in patients demonstrating treatment-responsive clinical stabilization (1). Based on the predefined protocol criteria, ALPA-treated patients received a mean of 2.68 artificial liver support sessions per patient, with a mean inter-treatment interval of 1.1 days. Anticoagulation with argatroban (loading dose 0.13 mg/kg; maintenance 0.03 mg/kg/h) was administered, with dosage adjustments based on activated partial thromboplastin time (APTT) and transmembrane pressure monitoring. The mean treatment duration was 166.7 min per session (Figure 1).

### Statistical analysis

Statistical analyses were performed using SPSS 26.0 (IBM Corp., Armonk, NY) and GraphPad Prism 10.0 (GraphPad Software, San Diego, CA) for data analysis and visualization. Continuous variables with normal distribution were presented as mean ± standard deviation (SD), while non-normally distributed data were expressed as median (interquartile range, IQR). Between-group comparisons were conducted using independent samples t-test for normally distributed data or Mann–Whitney U test for non-parametric data. Paired samples t-test was employed for within-group comparisons before and after treatment. Categorical variables were summarized as counts (percentages) and analyzed using chi-square test or Fisher’s exact test as appropriate. For time-to-event data, Kaplan–Meier survival analysis and Cox proportional hazards regression models were employed.

## Results

### Pathogen composition

**Figure 2:**
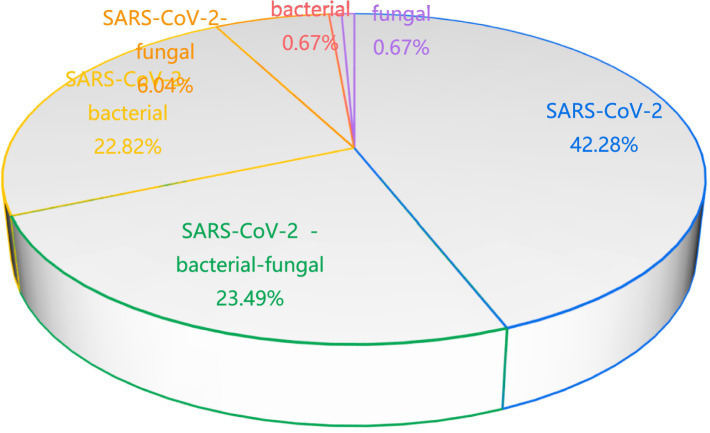
Pathogen composition.

SARS-CoV-2 monoinfection was the most prevalent (42.28% of total cases). Coinfections were observed in 52.35% cases, including: SARS-CoV-2 -bacterial-fungal (23.49%), SARS-CoV-2-bacterial (22.82%), SARS-CoV-2-fungal (6.04%). Non-viral infections were less frequent (4.70% collectively): Bacterial or fungal monoinfection (0.67%), Bacterial-fungal coinfection (4.03%) (Figure 2).

### Comparative analysis of baseline characteristics and laboratory parameters pre- and post-treatment in both study groups

Study participants were stratified by ALPA intervention status: Control Group (*n* = 95) versus ALPA Group (*n* = 56). Comparative analyses of baseline characteristics and longitudinal laboratory profiles were performed between groups. No significant intergroup differences were observed in baseline demographics (sex, age, height, weight) or PSI, nor in laboratory parameters (WBC, CRP, PCT, IL-6) (all *p* > 0.05). Pre-treatment LY% were significantly higher in the Control group versus ALPA group (*p* = 0.023). Post-treatment analysis revealed significantly lower levels of WBC, LYM, CRP, PCT and IL-6 in ALPA group compared to controls (all *p* < 0.01) (Tables 1, 2).

**Table 1:** Comparison of baseline characteristics, laboratory indicators and PSI Score of pre-treatment between the two groups (*n* = 151).

Outcome	ALPA group (*n* = 56)	Control group (*n* = 95)	T/Z/x2	*p*-value
Gender			0.125	0.724
Male	41 (73.2%)	67 (70.5%)		
Female	15 (26.8%)	28 (29.5%)		
Age (year)	70.59 ± 14.033	73.50 ± 14.693	1.207	0.23
Hight (cm)	168.00 (161.25–173.50)	170.00 (162.25–172.75)	−0.376	0.707
Weight (kg)	65.57 ± 15.190	68.12 ± 15.593	0.706	0.483
WBC (*10^9/L)	8.45 ± 6.292	11.21 ± 13.332	1.72	0.088
LY (%)	0.40 (0.20–0.60)	1.10 (0.40–5.20)	−5.354	<0.001
CRP (mg/L)	64.85 (22.53–138.98)	64.00 (23.90–136.00)	−0.112	0.911
PCT (ng/mL)	0.34 (0.12–1.60)	0.54 (0.22–3.52)	−1.621	0.105
IL-6 (pg/mL)	49.97 (4.63–116.73)	33.23 (13.30–118.22)	−0.157	0.876
PSI score	147.04 ± 45.020	144.28 ± 35.655	−0.415	0.679

**Table 2:** Comparison of laboratory indicators and PSI Score of post-treatment between the two groups (*n* = 151).

Outcome	ALPA group (*n* = 56)	Control group (*n* = 95)	T/Z	*p*-value
WBC (*10^9/L)	9.39 ± 6.190	12.81 ± 11.154	2.103	0.037
LY (%)	0.30 (0.10–0.78)	1.20 (0.40–5.58)	−4.319	<0.001
CRP (mg/L)	17.05 (0.00–50.68)	56.10 (16.20–178.70)	−3.877	<0.001
PCT (ng/mL)	0.62 (0.20–2.24)	1.24 (0.30–4.30)	−2.197	0.028
IL-6 (pg/mL)	11.06 (3.12–101.03)	37.75 (16.56–236.22)	−2.728	0.006

Longitudinal changes in laboratory indicators were analyzed using paired t-tests. Cases with missing values or significant outliers were excluded from the analysis, with the final sample sizes detailed in Table 3. In the Control group, post-treatment analysis showed a significant increase in WBC count (*p* < 0.05), while LYM, PCT, CRP, and IL-6 levels remained unchanged (*p* ≥ 0.05) (Table 3). The ALPA group demonstrated significant reductions in CRP and IL-6 levels (*p* < 0.001), with no significant changes observed in WBC, LYM, or PCT levels (*p* ≥ 0.05) (Table 4).

**Table 3:** Changes in laboratory indicators pre- and post-treatment in the control group.

Laboratory indicators	Pre-treatment	Post-treatment	Mean difference (95%CI)	df	t	*p*-value
WBC (*10^9/L)	10.09 ± 5.951	12.37 ± 10.366	−2.28 (−4.523--0.033)	90	−2.016	0.047
LY (%)	3.60 ± 6.101	4.67 ± 7.507	−1.07 (−2.628–0.483)	90	−1.37	0.174
CRP (mg/L)	80.81 ± 72.153	98.16 ± 96.255	−17.34 (−39.521–4.840)	83	−1.555	0.124
PCT (ng/mL)	2.23 ± 3.616	2.50 ± 3.834	−0.28 (−1.534–0.974)	57	−0.447	0.657
IL-6 (pg/mL)	101.56 ± 119.574	534.34 ± 1937.890	−432.78 (−1241.954–376.394)	24	−1.104	0.281

**Table 4:** Changes in laboratory indicators pre- and post-treatment in the ALPA group.

Laboratory indicators	Pre-treatment	Post-treatment	Mean difference (95%CI)	df	t	*p*-value
WBC (*10^9/L)	8.45 ± 6.292	9.39 ± 6.190	−0.94 (−2.502–0.623)	54	−1.205	0.233
LY (%)	0.52 ± 0.573	0.56 ± 0.566	−0.05 (−0.214–0.119)	45	−0.579	0.565
CRP (mg/L)	90.68 ± 81.873	44.17 ± 62.400	46.51 (24.166–68.848)	55	4.174	<0.001
PCT (ng/mL)	2.89 ± 7.247	1.55 ± 2.318	1.34 (−0.586–3.267)	41	1.404	0.168
IL-6 (pg/mL)	62.25 ± 69.185	534.34 ± 1937.890	52.72 (24.154–81.277)	23	3.818	<0.001

### Mortality comparison between groups

Patients who died during the first hospitalization or were DAMA were classified as mortality cases, whereas those with clinical improvement and successful discharge were categorized as survival cases for mortality rate calculation. As shown in the Table 5, the overall mortality rate was 66.9%. In the control group (*n* = 95), 18 patients improved while 77 died, yielding a mortality rate of 81.1%. In contrast, the ALPA group (*n* = 56) showed 32 improved cases and 24 deaths, corresponding to a mortality rate of 42.9%. The ALPA group demonstrated significantly lower mortality compared to the control group, with a 38.2% reduction in mortality rate (χ^2^ = 23.207, *p* < 0.001) (Table 5).

**Table 5:** Comparison of mortality rate between the two groups.

Outcome	Control group (*n* = 95)	ALPA group (*n* = 56)	X2	*p*-value
Death	77 (81.1%)	24 (42.9%)	23.207	<0.001
Clinical improvement	18 (18.9%)	32 (57.1%)		

### Factors influencing survival time and prognostic outcomes

Cases with incomplete survival data (e.g., missing treatment initiation dates or undefined death times) were excluded from Kaplan–Meier survival analysis and Cox regression modeling. The final cohort comprised 41 controls and 55 ALPA-treated patients. In contrast to the preceding analysis, survival time was calculated from study entry until either patient death or the last documented follow-up (including cases lost to follow-up). Mortality events were defined as all-cause death or DMAM occurring during the follow-up period (not during the first hospitalization). To identify prognostic factors in severe pneumonia patients, we initially conducted univariable Cox regression analysis. As shown in Table 6, univariable Cox regression identified CRP (HR = 1.004, *p* = 0.007), PSI score (HR = 1.008, *p* = 0.010), and ALPA treatment (HR = 4.398, *p* < 0.001) as significant prognostic factors. Multivariable analysis demonstrated that PSI score (HR = 1.009, *p* = 0.006) and ALPA treatment (HR = 4.134, p < 0.001) remained independently associated with survival outcomes. No laboratory indicators showed independent prognostic significance in the multivariable analysis (All *p* > 0.05).

**Table 6:** Analysis of independent risk factors for survival outcomes.

	Univariable cox regression		Multivariable cox regression	
Variables	*p*-value	HR (95%CI)	*p*-value	HR (95%CI)
Age (year)	0.194	1.013 (0.993–1.033)		
Gender	0.253	1.444 (0.769–2.709)		
WBC (*10^9/L)	0.210	1.028 (0.985–1.072)		
LY (%)	0.104	1.028 (0.994–1.062)		
CRP (mg/L)	0.007	1.004 (1.001–1.007)	0.216	1.002 (0.999–1.005)
PCT (ng/mL)	0.587	0.978 (0.904–1.059)		
IL-6 (pg/mL)	0.173	1.002 (0.999–1.005)		
PSI score	0.010	1.008 (1.002–1.013)	0.006	1.009 (1.002–1.015)
ALPA treatment	<0.001	4.398 (2.568–7.529)	<0.001	4.134 (2.404–7.110)

**Figure 3:**
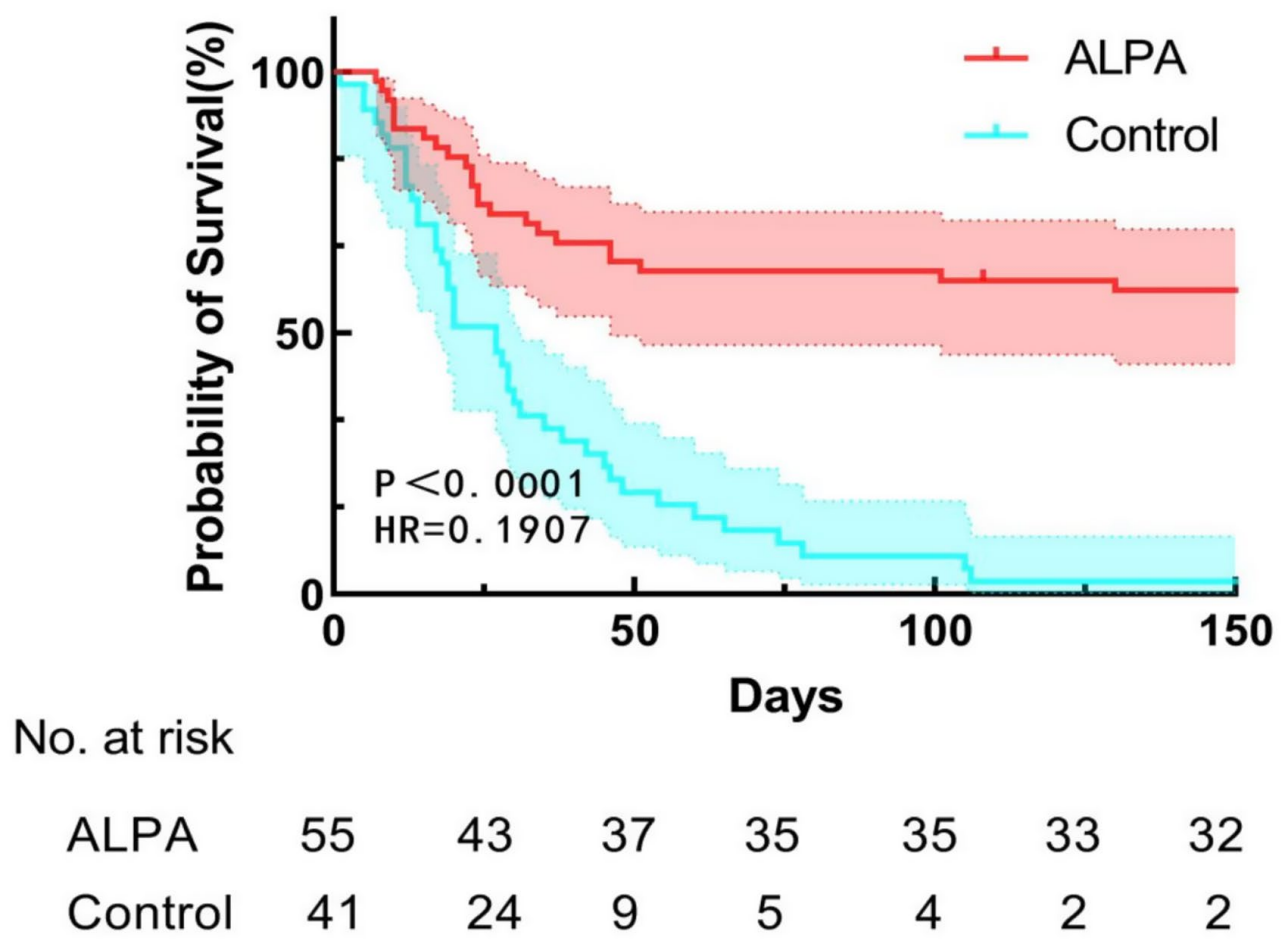
Kaplan–Meier estimates of overall survival in patients receiving ALPA versus standard therapy.

Survival analysis was performed using the Kaplan–Meier method, with comparisons between groups assessed by the log-rank test. Survival curves are presented in Figure 3. The ALPA group demonstrated prolonged survival, with a mean survival time of 95.2 days (95% CI: 77.9–112.5) and median survival not reached (follow-up cutoff: <50% events). The non-ALPA group had significantly shorter median (27.0 days, 95% CI: 17.6–36.4) and mean (34.4 days, 95% CI: 24.9–43.9) survival times (HR = 0.191, *p* < 0.0001).

## Discussion

Severe pneumonia in elderly patients represents a significant clinical challenge due to its rapid progression, complex manifestations, and persistently high mortality rates. These patients frequently develop acute respiratory failure, often complicated by cytokine storms, septic shock, and multiple organ dysfunction syndrome (MODS), posing substantial therapeutic challenges (2–4). The progression to critical illness can be attributed to multiple factors: compromised general condition, functional disability, malnutrition, diminished cough and gag reflexes, immune dysfunction, impaired pulmonary function, and moderate airflow limitation. Common comorbidities include respiratory and neurological disorders, along with other underlying conditions (e.g., diabetes, coronary artery disease, malignancies, Parkinson’s disease, GERD), and concurrent medications (e.g., corticosteroids, chemotherapeutic agents). Furthermore, clinical presentations are often atypical. Elderly pneumonia frequently presents with nonspecific symptoms such as anorexia, altered mental status, or exacerbation of pre-existing conditions, rather than characteristic respiratory symptoms (e.g., high fever, productive cough), leading to frequent underdiagnosis and treatment delays. Additionally, these patients are particularly susceptible to secondary bacterial and fungal infections following viral illnesses, along with associated antimicrobial resistance issues (11, 12). Severe pneumonia may result from bacterial, viral, fungal or mixed infections. The cytokine storm (CS) represents a key pathophysiological feature that contributes significantly to disease progression to ARDS and MODS in severe pneumonia cases (13).

ALPA demonstrates superior efficacy in treating severe pneumonia, especially in cases complicated by sepsis, ARDS, or multi-organ failure. The key advantage of this approach lies in its selective removal of inflammatory mediators while preserving essential coagulation factors and beneficial proteins. Additionally, it eliminates the need for plasma exchange-related blood product dependence and prevents the loss of albumin and globulin. In contrast to continuous renal replacement therapy (CRRT) with limited small-molecule clearance, extracorporeal membrane oxygenation (ECMO) lacking clearance function, and plasma exchange causing nonselective plasma loss, ALPA provides targeted immunomodulation during cytokine storms (14, 15). Owing to its excellent compatibility, ALPA can be combined with CRRT or ECMO to establish an integrated multi-organ support system, demonstrating particular efficacy in managing sepsis-induced liver injury and fulminant cytokine release syndrome.

Our study revealed a mean onset age of 72.41 in elderly patients. The cohort comprised predominantly geriatric populations, with octogenarians (*n* = 86, 56.9%) and septuagenarians (*n* = 112, 74.17%) accounting for the majority. The increased vulnerability in elderly populations is primarily attributed to age-related functional decline, compromised immunity, and multiple comorbidities (1). Male predominance was observed (108 cases, 71.5%), consistent with previous studies (16). This gender disparity may be associated with higher smoking prevalence in males compared to females.

This study found that viral infections accounted for the highest proportion (42.28%), all of which were caused by the novel coronavirus, suggesting it remains the primary viral agent of severe pneumonia in elderly patients. Mixed viral-bacterial-fungal co-infections were prevalent (56.38%), aligning with prior findings (12). In contrast, isolated bacterial or fungal infections were rare (0.67% each). The mortality rate for isolated viral infections was 42.8%, rising to 86.7% in cases of mixed infections. These findings suggest that preventing viral infections—through vaccination and protective measures—is critical to reducing severe pneumonia risk in elderly populations. Upon viral infection onset, prompt measures to prevent secondary bacterial or fungal infections are essential to mitigate mortality (12).

In this study, the mean PSI scores for the ALPA and the control groups were no significant statistical difference (147.04 ± 45.020 vs. 144.28 ± 35.655, *p* > 0.05), indicating that the two groups were equally severe and comparable.

The two groups showed comparable baseline characteristics in gender, age, height, weight, WBC, CRP, PCT, and IL-6 levels (all *p* > 0.05). Post-treatment analysis revealed no statistically significant differences in WBC, LY%, or PCT levels between groups (*p* > 0.05). Comparative analysis of pre- and post-treatment biomarker levels revealed: in the control group, neither CRP nor IL-6 levels changed significantly (*p* > 0.05), with WBC showing an overall increase; conversely, the ALPA group exhibited significant reductions in both CRP and IL-6 (*p* < 0.001). The control group had mean IL-6 concentrations 56-fold higher than the ALPA group.

Cytokine storm syndrome (CSS) is a life-threatening systemic inflammatory syndrome characterized by excessive cytokine release and immune hyperactivation, triggered by infections (viral/bacterial), malignancies, or autoimmune disorders (6, 17). CSS manifestations were first documented in SARS and MERS-CoV outbreaks, with subsequent reports in H1N1, H7N9, and other severe viral infections (2, 18). Notably, CSS correlates with high mortality rates, a pattern later confirmed in COVID-19 patients (19, 20). IL-6 stimulates hepatocytes to synthesize acute-phase proteins, including CRP and serum amyloid A (SAA) (21). In CSS, these acute-phase proteins amplify inflammatory cascades via complement activation and leukocyte recruitment, ultimately leading to tissue injury (22). *In vitro* adsorption assays demonstrated significantly higher cytokine binding affinity in phosphate-buffered saline versus plasma, with activated charcoal and polystyrene resins showing the most pronounced differences (23). The resin exhibited differential cytokine clearance efficiency, with optimal removal observed for TNF-*α*, IFN-α and IL-6. In vitro perfusion studies demonstrated that the resin achieved TNF-α clearance of 32.5% ± 3.5% and IL-6 removal of 71.4% ± 3.8% following 4-h perfusion. The cytokine removal capacity of resin adsorption likely involves its high-affinity binding to cytokine-albumin complexes in plasma (24). Resin demonstrates high albumin-binding affinity. During blood perfusion, leukocyte activation within the filter triggers cytokine release. These cytokines, due to their high molecular weight and strong binding affinity for plasma proteins, are subsequently removed by adsorption. A clinical study by Yi et al. (25) demonstrated significant reductions in IL-6, IL-8, and TNF-*α* following hemoperfusion therapy in 20 liver failure patients (all *p* < 0.05). Artificial liver systems attenuate cytokine storm through IL-6 removal, consequently suppressing CRP production (6, 23). Results from Tables 2, 3 indicate that conventional comprehensive treatment in elderly patients with severe pneumonia shows limited efficacy in rapidly removing cytokines (e.g., IL-6). In contrast, artificial liver plasma adsorption therapy demonstrates rapid clearance of inflammatory cytokines and their associated acute-phase proteins (e.g., CRP). This intervention may help mitigate CS and prevent rapid disease progression (6, 23, 26). Neither group demonstrated significant fluctuations in PCT levels, consistent with the observations by Zhong et al. (27). This finding corroborates current artificial liver therapy guidelines for cytokine storm management (6), which do not recommend PCT monitoring. The observed phenomenon may be attributed to two key factors. First, this study exclusively enrolled critically ill ICU patients, among whom secondary bacterial and fungal infections commonly developed following viral infection. Second, the resin-based material used in the artificial liver plasma adsorption system demonstrates strong albumin-binding affinity and predominantly anionic properties. In contrast, PCT circulates primarily in free form with limited albumin binding capacity and exhibits weakly cationic characteristics. This fundamental physicochemical mismatch likely accounts for the lack of significant PCT reduction.

The results of this study demonstrated a 38.2% reduction in mortality (81.1% vs. 42.9%, *p* < 0.001). Cox regression demonstrated that admission infection-related markers were not significant prognostic factors for clinical outcomes or survival duration. In contrast, artificial liver plasma adsorption (ALPA) therapy showed significant survival benefits in our cohort. While CRP did not emerge as an independent prognostic factor in multivariable Cox regression, univariate analysis suggested its potential association with clinical outcomes. Importantly, ALPA therapy effectively reduces CRP levels. ALPA therapy exerts multiple therapeutic effects by rapidly clearing inflammatory mediators, restoring immune homeostasis, and preventing immune-mediated tissue injury. These mechanisms collectively ameliorate multiorgan dysfunction (including hepatic/renal impairment and respiratory failure) and facilitate organ recovery. We therefore recommend ALPA therapy for elderly patients with severe pneumonia, unless contraindicated by severe active bleeding or hemodynamic instability, irrespective of baseline laboratory parameters.

Unlike prior survival studies investigating artificial liver blood purification in COVID-19 patients (9) that mainly employed plasma exchange, our research specifically examined plasma adsorption therapy. This method eliminates the need for plasma resources required by exchange techniques, significantly improving clinical applicability. Additionally, we conducted a comparative mortality analysis of secondary mixed infections involving bacterial, fungal, and viral pathogens. This extends the therapeutic scope of artificial liver blood purification from viral infections to cytokine storms caused by bacterial and fungal infections. However, this study has several limitations. It is a single-center retrospective analysis with a limited sample size, and the findings warrant validation through large-scale, randomized controlled trials (RCTs) focusing on severe pneumonia in the elderly population. Future research should focus on large-scale, multicenter, prospective studies to evaluate the efficacy of ALPA in treating CSS-related diseases, including severe/critical pneumonia in elderly patients. Such studies would not only validate this novel therapeutic approach but also expand treatment options and improve clinical outcomes for this vulnerable population.

## Data Availability

The raw data supporting the conclusions of this article will be made available by the authors, without undue reservation.
